# A decade of immune-checkpoint inhibitors in cancer therapy

**DOI:** 10.1038/s41467-020-17670-y

**Published:** 2020-07-30

**Authors:** Caroline Robert

**Affiliations:** Gustave Roussy and Paris-Saclay University, 94800 Villejuif, France

**Keywords:** Cancer immunotherapy, Tumour immunology

## Abstract

Immunotherapy using immune-checkpoint modulators revolutionizes the oncology field far beyond their remarkable clinical efficacy in some patients. It creates radical changes in the evaluation of treatment efficacy and toxicity with a more holistic vision of the patient with cancer.

## The revival of tumor immunotherapy

The paramount achievement in cancer treatment in the last decade has undoubtedly been the introduction of T cell targeted immunomodulators blocking the immune checkpoints CTLA-4 and PD1 or PDL1. In 2011, ipilimumab, the first antibody blocking an immune checkpoint (CTLA4) was authorized. This was rapidly followed by the development of monoclonal antibodies targeting PD1 (pembrolizumab and nivolumab) and PDL1 (atezolizumab and durvalumab). Anti-PD1/PDL1 antibodies have become some of the most widely prescribed anticancer therapies. T-cell-targeted immunomodulators are now used as single agents or in combination with chemotherapies as first or second lines of treatment for about 50 cancer types. There are more than 3000 active clinical trials evaluating T cells modulators, representing about 2/3 off all oncology trials^1^.

Yet, ten years ago, just before the era of immune checkpoint inhibitors (ICI), solid tumor immunotherapy was in a grim situation. It was based on immunocytokines such as interleukin-2 or alpha-interferon that were poorly effective and highly toxic. Clinical research trials had tested diverse forms of cancer vaccines that were mostly ineffective^2^. Immunotherapy had a small and shrinking audience at international oncology meetings while sessions related to the new booming field of targeted therapy were overflowing. However, after the first success of ICI immunotherapy and until today, the situation has reversed, immunotherapy leads the field and immunologists have regained a major influence in cancer research as illustrated by the attribution of the 2018 Nobel Prize in Medicine to the two immunologists who were at the origin of the concept of ICI-based immunotherapy, James Allison and Tasuku Honjo^3^.

### A radically new vision of cancer management

This place of honor in the arena of cancer treatment is unquestionably well deserved owing to the immense clinical progress ICI brought about in the treatment of certain aggressive cancers such as metastatic melanoma, the first disease where ICI efficacy was demonstrated^4,5^. Far beyond its remarkable efficacy in some patients, ICI immunotherapy revolutionized the oncology field in more than one way. It has changed the way physicians evaluate treatment efficacy or manage adverse events. It also resulted in a more holistic view of cancer patients, beyond the mere cancer cells, and created new and fruitful interactions between immunologists, oncologists and other organ-specialists.

Indeed, the success of immunotherapy that relies on cancer destruction through the activation of the host immune system led to a more complete view of cancer. It now takes into account not only the cancer cells to be targeted and destroyed but also the cancer immune environment. We are now fully aware of the little relevance of usual preclinical testing of cancer drugs performed on cultured cancer cells lines and immune-compromised animals. The latter completely overlook the immune system. New and more reliable preclinical models using immune-competent animals are now more widely used.

New tools for translational and clinical research now include immune parameters such as the presence and activation status of tumor infiltrating T cells, expression of the immune checkpoint PDL1 or the evaluation of the tumor mutational burden (TMB)^6^. Interestingly, TMB, which represents the ratio of non-synonymous somatic mutations per tumor DNA megabase, was historically mostly associated with resistance to cytotoxic or targeted therapy. On the other hand, with ICI immunotherapy, the potential for multiple neoantigens originating from highly mutated tumors appears as a favorable factor for response^7^. This is why lung cancers of smokers, characterized by a high tobacco-induced genetic somatic mutations respond better to immunotherapy than the lower TMB-associated lung cancers from non-smoking patient^7^. The correlation between a high TMB and response to immunotherapy led to the authorization of anti-PD1 drugs for the highly mutated cancers linked to a mismatch DNA repair deficiency (microsatellite instability)^8^. This is a rare example in the history of cancer therapy that a drug was authorized based on a biological oncologic mechanism regardless of the underlying tumor type.

ICI immunotherapy can induce delayed tumor responses even after an initial increase in the size of the metastases. Such pseudo-progressions might be due to a delayed efficacy of the immunotherapy or to an initial recruitment of immune cells resulting in a transitory tumor increase in size. Thus, the usual standard radiologic evaluation criteria (RECIST-1.1), routinely applied to monitor responses to chemotherapies or targeted therapies, were not adapted to these new kinetics of responses. New guidelines for evaluation criteria, including an extended delay to confirm or disprove tumor increase, have been incorporated in the iRECIST (immune RECIST) evaluation system^9^.

We also have to modify the main end-points of the clinical trials evaluating ICI. The benefit of ICI is not properly captured by classical endpoints, such as median progression-free-survival, response rates or hazard ratio (HR), because ICI may have a delayed effect with a variable proportion of long term survivors (plateau or tail of the curve). Analyses of the proportion of patients who are alive or free of progression at late time-points (landmark analyses) or of the restricted mean survival time (measuring the average survival from time 0 to a specified time), are more adapted to ICI immunotherapy^10^.

Another profound change is related to the type of adverse events associated with immunotherapy^11^. Unsurprisingly they are radically different from those associated with previous treatments, cytotoxic or targeted therapies. Since ICI action mechanism relies on the inhibition of the physiological brake of immune activation, they often have off-target effects resulting in immune-mediated inflammation of diverse organs or tissues. A wide and whole new registry of iatrogenic effects, referred to as immune-mediated or immune related, can look like autoimmune diseases, such as autoimmune thyroiditis, eventually resulting in permanent hypothyroidism or inflammatory bowel diseases. They can sometimes be severe, especially when anti-CTLA and anti-PD1 are used in combination, with up to 60% of grade 3-5 adverse events. Although rare, ICI-related deaths may occur when severe iatrogenic event such as myocarditis, encephalitis, or acute hypophysitis are not readily diagnosed and treated with high dose steroids and more potent immunosuppressors^12^. This new spectrum of adverse events has required rapid and efficient interactions between treating oncologists and diverse organ specialists as well as internists in order to optimize the management of the wide range of immune related adverse events.

### A hope for cure but for a minority of patients

One of the most impressive successes of ICI has been long term remission in spite of treatment discontinuation, raising substantial hope for cure for some patients^13^. This is particularly well documented in melanoma patients who achieve a complete response, meaning a complete disappearance of all visible metastases. This is the case for about 20% of patients with melanoma treated with anti-PD1 with or without anti-CTLA-4. It is now widely accepted that treatment can be discontinued for such patients, after at least 6 months of therapy since their risk of relapse is estimated to be less than 10% over the 5 year follow-up that is available today^13^. Such long complete remission of the disease was totally unimaginable before the era of ICI. However, not all cancer types respond as well as melanoma and data on the possibility to discontinue therapy is not as mature for other cancers.

Still in melanoma, which leads the field for ICI development, one year of adjuvant treatment with anti-PD1 was shown to decrease the risk of relapse after surgical resection of regional lymph nodes metastases (stage III)^14,15^. In other cancer types, such as lung cancers, ICI are presently being evaluated as adjuvant therapies. One major change for patients and physicians stems for the fact that the impact of adverse events is not similar in patient with metastatic cancers or in those who receive an adjuvant treatment in the aim of decreasing a risk of relapse. In the latter situation, the possibility of inducing a severe or a permanent adverse effect has to be cautiously evaluated. For example, the risk of hypothyroidism that occurs in up to 10% of anti PD1 treated patients is considered acceptable in the context of a metastatic disease. In an adjuvant situation, this 10% risk of having to take substitutive hormonal treatment until the end of one’s life has to be balanced with the expected treatment benefit.

The attitude of the patients toward cancer immunotherapy is usually rather positive. The patients often appreciate the idea of fighting against cancer by mobilizing their own immune system. Because of this frequent adhesion to the treatment strategy, it is likely that the patients can be more actively involved in their treatment and that the interaction between patients and physicians can be facilitated at least at the phase of treatment initiation.

One counter effect is that immunotherapy is somewhat victim of its own success. Attractiveness of this treatment strategy among patients and the general public, reinforced by the simplified and embellished media coverage, has set very high expectations and is a source of profound disappointment in patients for whom ICI treatment does not meet its promise, and they are still a majority.

## Conclusions

Finally, it took a long time for immunotherapy to penetrate a circle of active cancer drugs. It is finally the case with ICI that were developed and authorized for several cancer types with an unprecedented speed over the last decade. In spite of a huge step forward, ICI has not resolved the issue of cancer treatment. With immune-checkpoint immunotherapy, a door has been opened, but the case is not closed. Our hopes for the next decade are that biomarkers for predicting ICI efficacy and toxicity will be identified together with pharmacodynamics parameters to optimize ICI regimens and new combinations.

## References

[1] Xin Y J, Hubbard-Lucey VM, Tang J (2019). Immuno-oncology drug development goes global. Nat. Rev. Drug Discov..

[2] Rosenberg SA, Yang JC, Restifo NP (2004). Cancer immunotherapy: moving beyond current vaccines. Nat. Med..

[3] Ledford H, Else H, Warren M (2018). Cancer immunologists scoop medicine Nobel prize. Nature.

[4] Robert C (2015). Pembrolizumab versus ipilimumab in advanced melanoma. N. Engl. J. Med..

[5] Larkin, J. et al. Five-year survival with combined nivolumab and ipilimumab in advanced melanoma. *N. Engl. J. Med*. 10.1056/NEJMoa1910836 (2019). 10.1056/NEJMoa1910836 31562797

[6] Blank CU, Haanen JB, Ribas A, Schumacher TN (2016). CANCER IMMUNOLOGY. The ‘cancer immunogram’. Science.

[7] Schumacher TN, Scheper W, Kvistborg P (2019). Cancer neoantigens. Annu. Rev. Immunol..

[8] Le DT (2015). PD-1 blockade in tumors with mismatch-repair deficiency. N. Engl. J. Med..

[9] Seymour L (2017). iRECIST: guidelines for response criteria for use in trials testing immunotherapeutics. Lancet Oncol..

[10] Liang F, Zhang S, Wang Q, Li W (2018). Treatment effects measured by restricted mean survival time in trials of immune checkpoint inhibitors for cancer.. Ann. Oncol..

[11] Boutros C (2016). Safety profiles of anti-CTLA-4 and anti-PD-1 antibodies alone and in combination. Nat. Rev. Clin. Oncol..

[12] Wang DY (2018). Fatal toxic effects associated with immune checkpoint inhibitors: a systematic review and meta-analysis. JAMA Oncol..

[13] Robert C (2018). Durable complete response after discontinuation of pembrolizumab in patients with metastatic melanoma.. J. Clin. Oncol..

[14] Weber, J. et al. Adjuvant nivolumab versus ipilimumab in resected stage III or IV melanoma. *N. Engl. J. Med*. 10.1056/NEJMoa1709030 (2017). 10.1056/NEJMoa1709030 28891423

[15] Eggermont AMM (2018). Adjuvant pembrolizumab versus placebo in resected stage III melanoma. N. Engl. J. Med..

